# Detoxifying and depolymerizing microorganisms reveal intertwined guild collaborations in the gut microbiome of the generalist macro-algivorous fish *Kyphosus cinerascens*

**DOI:** 10.1128/mbio.03382-25

**Published:** 2026-06-24

**Authors:** Alvaro M. Plominsky, Aaron Oliver, Carlos Henriquez-Castillo, Sheila Podell, Jeremiah J. Minich, Simona Augyte, Jennica Lowell-Hawkins, Neil A. Sims, Eric E. Allen

**Affiliations:** 1 Center for Marine Biotechnology and Biomedicine,Scripps Institution of Oceanography, University of California San Diego 8784, La Jolla, California, USA; 2 Escuela de Medicina, Facultad de Ciencias de la Salud, Universidad del Alba 70015 , La Serena, Chile; 3 Department of Molecular Biology, School of Biological Sciences, University of California San Diego 315228, La Jolla, California, USA; 4 Department of Biology, Baylor University 14643, Waco, Texas, USA; 5 Ocean-Era, Inc., Kailua-Kona, Hawaii, USA; Georgia Institute of Technology, Atlanta, Georgia, USA

**Keywords:** fish gut microbiome, polyphenol utilization, polysaccharide utilization, sulfatases, macroalgal digestion

## Abstract

The biotransformation of macroalgal biomass represents a major catabolic challenge due to its structurally diverse polysaccharides and inhibitory polyphenols. Unlike terrestrial lignocellulosic substrates, macroalgal polysaccharides contain multiple monomer types, branching patterns, and sulfation states. Additionally, toxic macroalgal polyphenols have been shown to inhibit both microbial growth and their catalytic enzymes. While herbivorous fishes have evolved specialized gut microbiota to process these substrates, the enzymatic pathways remain poorly characterized, with few experimentally validated polysaccharide utilization loci or biochemically defined marine sulfatases, and limited understanding of polyphenol degradation. Here, we developed *in vitro* microcosms, based on the gut microbiome of the generalist macro-algivorous fish *Kyphosus cinerascens*, to temporally resolve the activity of the microbial guilds involved in macroalgal polysaccharide and polyphenol transformation. First, parallel cDNA/DNA amplicon sequencing was employed to distinguish the natural active fraction from transient gut microbiome taxa that became inactive/dead after their ingestion. Four medium combinations were able to propagate between 96% and 99% of the active hindgut microbial families, reproducing the cooperative degradation dynamics observed *in vivo*. Metagenomic and metatranscriptomic profiling of these four optimized *in vitro* microcosms served as models to assess the stepwise functional successions occurring in the natural gut microbiome. Early Gammaproteobacteria expressed enzymes linked to polyphenol detoxification and alginate degradation, followed by Bacillota, Bacteroidota, and Verrucomicrobiota guilds targeting more recalcitrant sulfated polysaccharides and polyphenols. Together, these results identified temporal and taxonomic coordination as key features of macroalgal biomass deconstruction, providing an experimentally tractable model for discovering novel carbohydrate-active enzymes and elucidating poorly understood pathways of marine polyphenol degradation.

**IMPORTANCE:**

Seaweed represents a source of sustainable biomass for various applications, but scalable industrial methods struggle to break down seaweed biomass into intermediate products due to the complexity of its constituents. Fish of the genus *Kyphosus* feed on different seaweed types by leveraging gastrointestinal bacteria to neutralize inhibitory polyphenols and convert their polysaccharides into simple sugars. This study identifies microbial groups that are transcriptionally active in natural fish hindgut microbiomes and how to propagate these active microbial communities *in vitro*. This enabled assessing how distinct microbial guilds act in succession to transform complex polysaccharides and polyphenols. Notably, this is the first study to assess the biotransformation capacities of macroalgal polyphenols by complex *in vitro* hindgut microbiomes of a generalist herbivorous fish. These findings advance our ecological understanding of cooperative degradation in marine gut symbioses and establish a tractable platform for discovering new enzymes and pathways with potential applications in algal biomass utilization.

## INTRODUCTION

The biotransformation of macroalgal biomass represents a major catabolic challenge due to the complexity of its polysaccharides (1), with diverse monomer types, branching patterns, sulfation states, and the presence of toxic polyphenols capable of inhibiting enzymes and microbial growth (2–4). These features distinguish macroalgal biomass from terrestrial lignocellulosic substrates and demand highly coordinated enzymatic strategies for effective biotransformation.

Marine herbivorous fishes have evolved specialized gut microbiota that enable them to neutralize the chemical defenses and digest the structurally complex polysaccharides of macroalgae. The carbohydrate-active enzymes (CAZymes) and sulfatases that mediate this process are distributed across multiple microbial taxa. Individual genomes lack the complete enzymatic repertoire to fully depolymerize macroalgae sulfated polysaccharides by themselves, underscoring the need for cooperative extracellular degradation (5–7). Such coordination is often encoded within polysaccharide utilization loci (PULs), which combine hydrolytic enzymes, sulfatases, transporters, and regulators. Yet, experimentally validated PULs for marine polysaccharides remain scarce, with less than 4% of known clusters linked to specific substrates (8). Likewise, the diversity of sulfatase enzymes required to cleave polysaccharide sulfate esters is only beginning to be appreciated, with just a handful of marine carbohydrate-active sulfatases biochemically characterized to date (9).

These gaps limit our capacity to predict pathway completeness *in silico*, especially for the most structurally complex substrates. Moreover, the presence of polyphenols adds another layer of complexity. While they function as secondary metabolites that protect macroalgae from herbivory and oxidative stress, their microbial degradation pathways remain poorly understood (2). Databases such as CAMPER (10) are only beginning to capture the diversity of human gut microbiome genes involved in polyphenol metabolism (11, 12), leaving open questions about how these compounds shape microbial succession and enzymatic activity in the gut environment of marine hosts. Investigating these interactions is essential, as the detoxification of polyphenols likely constrains the accessibility of underlying polysaccharides and mediates broader community dynamics during macroalgal digestion (2).

*Kyphosus* fish provide a natural model system to address these knowledge gaps. These fishes consume a wide variety of red, green, and brown macroalgae (13–15) and play key ecological roles in coral reef systems by regulating algal cover and mediating coral-algal competition (16). Previous studies have taxonomically characterized (15, 17) and laid the genomic foundation for the capacities of the *Kyphosus* gut microbiota to biotransform a variety of macroalgae sources (5, 7, 18). These metagenomic studies, assessing principally the distal end (hindgut) section of the host intestine, have revealed that the *Kyphosus* microbiomes are enriched in novel CAZyme and sulfatase families not found in terrestrial herbivores (5), and that many of these enzymes are phylogenetically distinct from known sequences in public databases (6). Moreover, recent *in vitro* cultivation efforts have offered a glimpse into the advantages of using a reproducible experimental setting to study these microbiomes (6). Overall, these studies have shown that degradation of macroalgae by the *Kyphosus* microbiomes is carried out by taxonomically diverse groups, most prominently members of the Bacteroidota and Bacillota phyla, that partition enzymatic functions in a stepwise division of labor (5–7, 18).

Together, these findings highlight both the ecological and biotechnological importance of *Kyphosus* gut symbionts. Ecologically, they illustrate how gut microbes underpin the ability of herbivorous fishes to utilize diverse macroalgal diets and thereby shape reef ecosystems. Biotechnologically, they offer a largely untapped resource for novel enzymes and microbial communities with potential applications in bioenergy, bioremediation, and seaweed-based aquaculture feeds (6). Yet, while genomic and compositional insights have expanded rapidly, relatively few studies have integrated longitudinal transcriptional data or experimentally propagated whole communities to capture active enzymatic processes under controlled conditions.

This study identified the transcriptionally active members of the *Kyphosus cinerascens* hindgut microbiota, a fish species with a generalist herbivorous feeding ecology encompassing green, brown, and red macroalgae. To propagate these active communities under controlled conditions, we screened different culture media and established *in vitro* microcosms (Fig. S1), from which we collected paired 16S rRNA amplicon, shotgun metagenomic, and shotgun metatranscriptomic sequencing data across temporal stages of algal biomass degradation. Together, these data provide unprecedented resolution into the stepwise, collaborative processes underlying macroalgal degradation in herbivorous fish and establish a reproducible experimental platform for exploring microbial succession, enzyme functionality, and community-level strategies for polysaccharide and polyphenol biotransformation.

## RESULTS AND DISCUSSION

### Amplicon-based profiling of natural and microcosm enrichments

**Fig 1:**
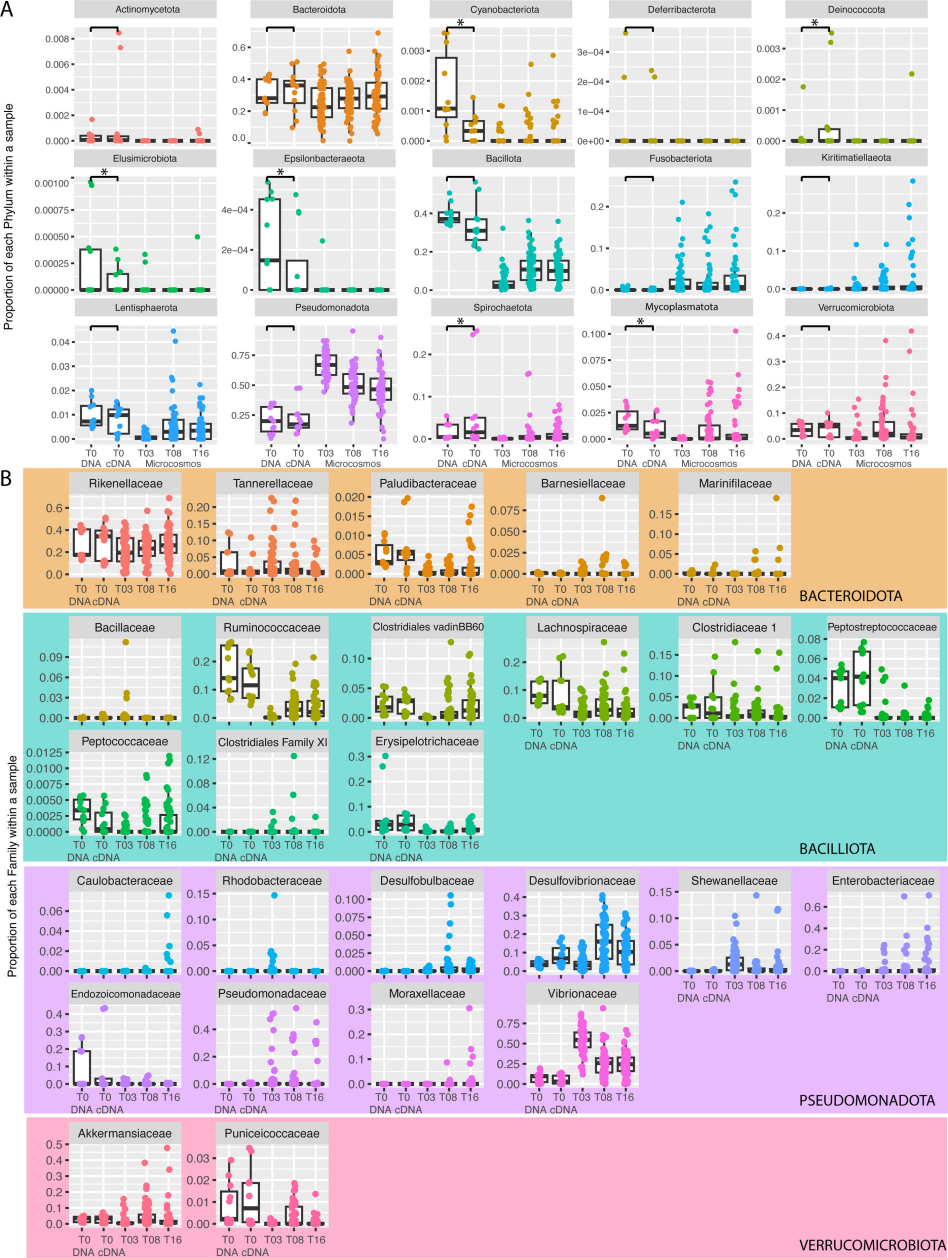
Proportional abundance of natural and propagated microbial communities of the *Kyphosus cinerascens* hindgut microbiome at (**A**) phylum- and (**B**) family-level taxonomic resolution. *X*-axis positions in each graph denote total DNA and cDNA fractions at the time of inoculation (T0), followed by results at 3, 8, and 16 days post-inoculation (T03, T08, and T16). Significant differences (*P* < 0.05) of mean abundances between natural *K. cinerascens* DNA and cDNA samples for each taxonomic group based on Tukey’s test are marked with an asterisk. Family-level abundances for Fusobacteria, Kiritimatiellaeota, Lentisphaerota, and Spirochaetota are not shown because nearly all of their taxonomically assignable reads were classified as belonging to a single family (i.e., Fusobacteriaceae, Kiritimatiellaceae, Victivallaceae, and Brevinemataceae, respectively). Taxa shown represent only those present at levels greater than 5 × 10^−4^% of all taxonomically assignable reads in at least one sample.

Six *K. cinerascens* specimens were collected, euthanized, and hindgut lumen contents were aseptically recovered under anaerobic conditions. Parallel DNA and RNA were extracted from the same preserved hindgut samples, with RNA reverse-transcribed to cDNA prior to 16S rRNA gene amplicon sequencing, enabling direct comparison of total and transcriptionally active microbial communities (Fig. S1). In parallel, hindgut contents from the same six fish were combined, homogenized, and used as a common inoculum to establish anaerobic *in vitro* microcosms amended with different macroalgal- and host-associated medium combinations (Fig. S1). Active *K. cinerascens* hindgut microbiota potentially involved in macroalgae biotransformations were identified by comparing cDNA- and DNA-based 16S rRNA gene amplicon data sets, using a high-throughput “Fast-Extract” DNA extraction procedure. This approach distinguished between active taxa and transient microbiota members that were ingested along with dietary items but became inactive/dead during their passage through the GI tract. Both data sets were generated from the same six *K. cinerascens* hindgut samples that served as source inocula for all microcosms (Fig. S1). At the phylum level, amplicon sequence variants (ASVs) assigned to Cyanobacteriota (potentially representing chloroplasts from the ingested macroalgae) and Epsilonbacteraeota/Campylobacterota varied greatly between DNA and cDNA abundances, with active (cDNA) fractions representing less than half of total DNA levels (Tukey’s honestly significant difference test *P* < 0.05; Fig. 1A). The phyla Bacillota, Deinococcota/Deinococcus-Thermus, Elusimicrobiota/*Ca*. Termite Group 1, and Mycoplasmatota differences were less pronounced, but still statistically significant, with higher representation of their ASVs in the DNA versus cDNA fractions (Tukey’s honestly significant difference test *P* < 0.05; Fig. 1A). Spirochaetota had between 20% higher representation in the cDNA (active) compared to the DNA (transient) fractions (Tukey’s honestly significant difference test *P* < 0.05; Fig. 1A). These apparent abundance enrichments can be due to actual increases in abundance of a given organism or due to the depletion of all other ASVs in the sample.

ASVs assigned to microbial families that were highly represented in the cDNA (active) fractions were also found in the microcosms (Fig. 1B), although ASVs assigned to phylum Bacillota were generally less abundant in the microcosms than in the initial inocula. In contrast, *in vitro* cultivation conditions seem to have favored some Pseudomonadota families that became overrepresented at distinct, sporadic time points (Fig. 1B). These results were consistent with a previous study showing high expression levels of genes for polysaccharide biotransformation were in Bacteroidota and Bacillota representatives from the hindgut of another *Kyphosus* species (18).

The taxonomic compositions of microcosms across all medium combinations over 16 days of cultivation were compared to determine the extent to which native *K. cinerascens* hindgut microbiota were being propagated *in vitro*. Four different medium combinations (microcosm numbers 30, 36, 70, and 78; Fig. S1 and Table S2) propagated 96%–99% of all ASVs assignable at the family level (Fig. S4). Although breakaway diversity estimates indicated that less than one-third of this diversity was replicated all the way down to individual ASV levels (Fig. S3), 22 of the 25 microbial families comprising more than 5 × 10^−4^% of the total ASVs in at least one sample were effectively propagated in cultures from these four medium compositions (Fig. S4). One caveat is that several Pseudomonadota families that were not detected in the cDNA fraction of natural *K. cinerascens* hindgut microbiomes (i.e., Caulobacteraceae, Rhodobacteraceae, and Desulfobulbaceae) were detected within the total DNA fraction, perhaps being reactivated during *in vitro* cultivation (Fig. S4).

The Association Networks framework and subsequent redundancy analysis of ASV co-occurrence patterns were used to corroborate which microcosm media best propagated the natural hindgut taxa. A network of samples was generated based on the similarity of their ASV co-occurrence patterns (Pearson correlation coefficients ≥ 0.7) to identify strongly associated pairs. Then their modularity was assessed through Markov cluster (MCL) assignments of highly connected sample clusters that shared similar community structures. In this network, microcosm 78 formed the most connected cluster with the active (cDNA fraction) *K. cinerascens* hindgut microbiomes of fish 3, 4, and 6 (Cluster 1; Fig. S5A), indicating a strong compositional similarity. The correlation network guided the subsequent redundancy analysis, which tested how specific medium components contributed to the propagation of these complex microbial assemblages. The fact that microcosm 78, with such a complex medium composition, was the best in propagating the *K. cinerascens* hindgut active microbiome is consistent with the results showing that a combination of many of the medium components, rather than a single component by itself, was required to enable the propagation of the natural hindgut community majority (Fig. S5B).

The media selected for use in detailed microcosm analyses (Fig. S1) contained several different combinations of macroalgae. Microcosm 30 contained (brown) *Turbinaria* spp. plus various (red) coralline representatives. Microcosm 36 also included (green) *Ulva* spp. Microcosms 75 and 78 contained the most complex macroalgae mixtures, encompassing (brown) *Turbinaria* spp., *Halidrys* spp., and *Dictyota* spp., various (red) coralline representatives, and (green) *Ulva* spp. The main distinction between the media of microcosms 75 and 78 was the addition of propionic acid, casein, and butyric acid in the latter (Fig. S1).

### Metagenomic and metatranscriptomic profiling of natural and microcosm enrichments

**Fig 2:**
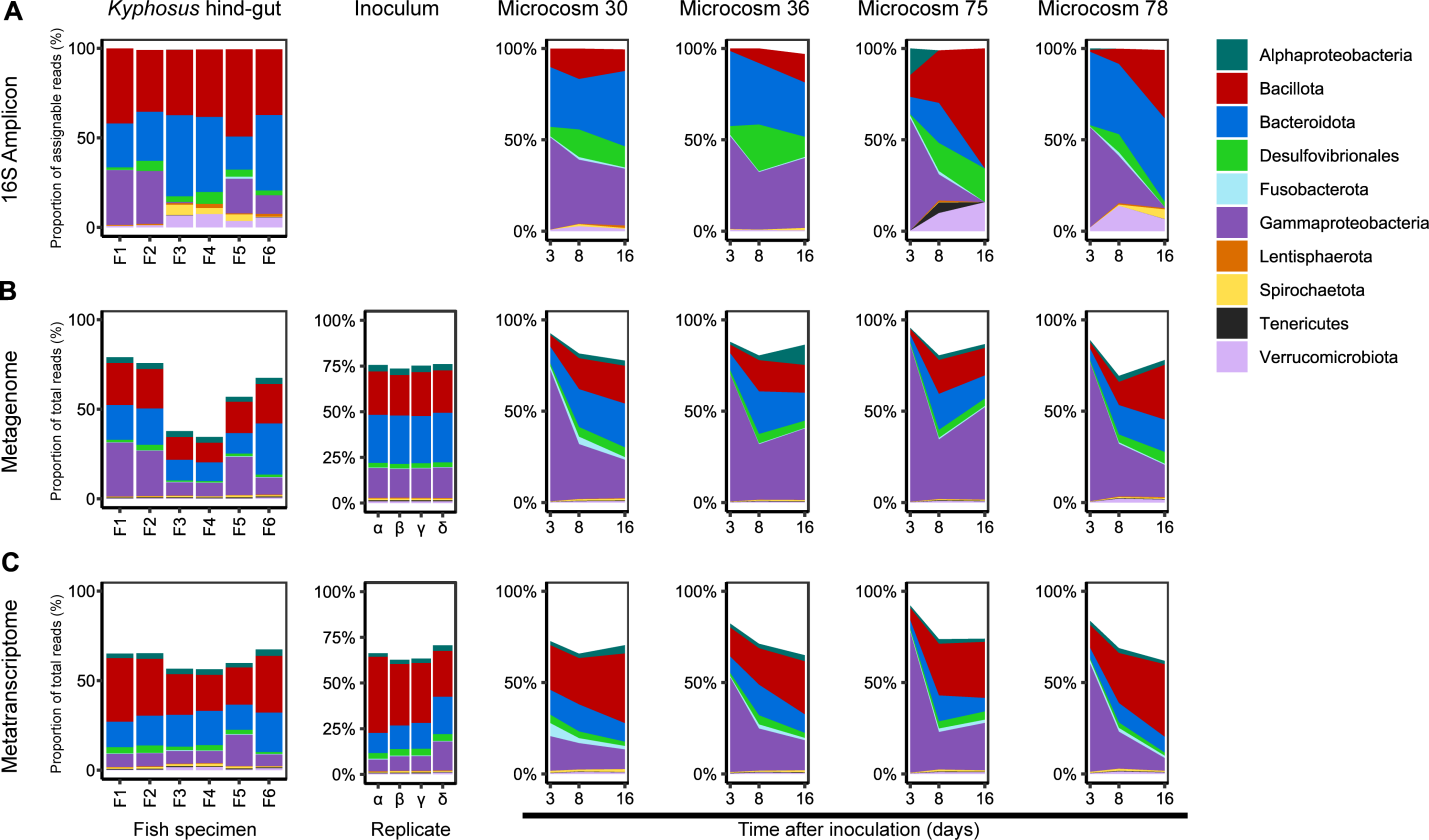
Overall composition and expression profile of microbial taxa from *K. cinerascens* natural hindgut, source inoculum, and selected *in vitro* microcosms. Taxonomic assignments of the microbial (**A**) 16S rRNA gene amplicon sequences, (**B**) metagenomic reads, and (**C**) metatranscriptomic reads from the microbial communities in six *K. cinerascens* natural hindgut samples, and four replicates of the inoculum mixture used to prepare all the microcosms. Three time points (3, 8, and 16 days after inoculation) are shown for the selected *in vitro* microcosms with the four culture conditions that propagated the largest number of microbial taxa from the active members of the natural hindgut samples.

The homogeneity of the combined *K. cinerascens* natural hindgut inoculum used to make all microcosms was corroborated by simultaneous metagenomic (Table S5) and metatranscriptomic analysis (Fig. 2B and C), which also confirmed the propagation of active microbial members from the natural *K. cinerascens* hindgut microbiota in the four selected microcosms (Fig. 2). However, as expected when comparing unassembled quality-filtered metagenomic and metatranscriptomic reads with 16S rRNA amplicon data derived from different nucleic acid extraction procedures (i.e., Fast-Extract versus a commercial Zymo kit), there were differences in the proportional abundances of certain groups. Such was the case for Alphaproteobacteria, which were underrepresented at the 16S rRNA gene level (Fig. 2A) but were prevalent across all time points of these four selected microcosms in both their proportional representation among metagenomic and metatranscriptomic reads (Fig. 2B and C). In contrast, the representation of Spirochaetota and Verrucomicrobiota was higher at the 16S rRNA gene level (Fig. 2A) compared to their proportional sequence-read representation assessed through metagenomic and metatranscriptomic approaches (Fig. 2B and C). The metagenomic data generated from the natural fish hindguts, the natural hindgut inoculum, and all three time points of the selected microcosms enabled the binning of 1,153 metagenome-assembled genomes (MAGs; Table S6). The propagation of populations from the main taxonomic groups comprising the natural *K. cinerascens* hindgut microbiome was further confirmed by the taxonomic redundancy observed between the MAGs generated from the source inoculum and the selected microcosms (Fig. S6).

The simultaneous extraction of DNA and RNA from the same biological material enabled the parallel comparison of the representation and transcriptomic profiles in the microbiomes from natural *K. cinerascens* hindgut and *in vitro* samples. To assess the expression of microbial enzymes that can be confidently associated with the biotransformation of large macroalgal polysaccharides, putative enzymes biotransforming green macroalgal polysaccharides (e.g., ulvan lyases) were excluded from this analysis because they could not be confidently connected to a single substrate (19).

**Fig 3:**
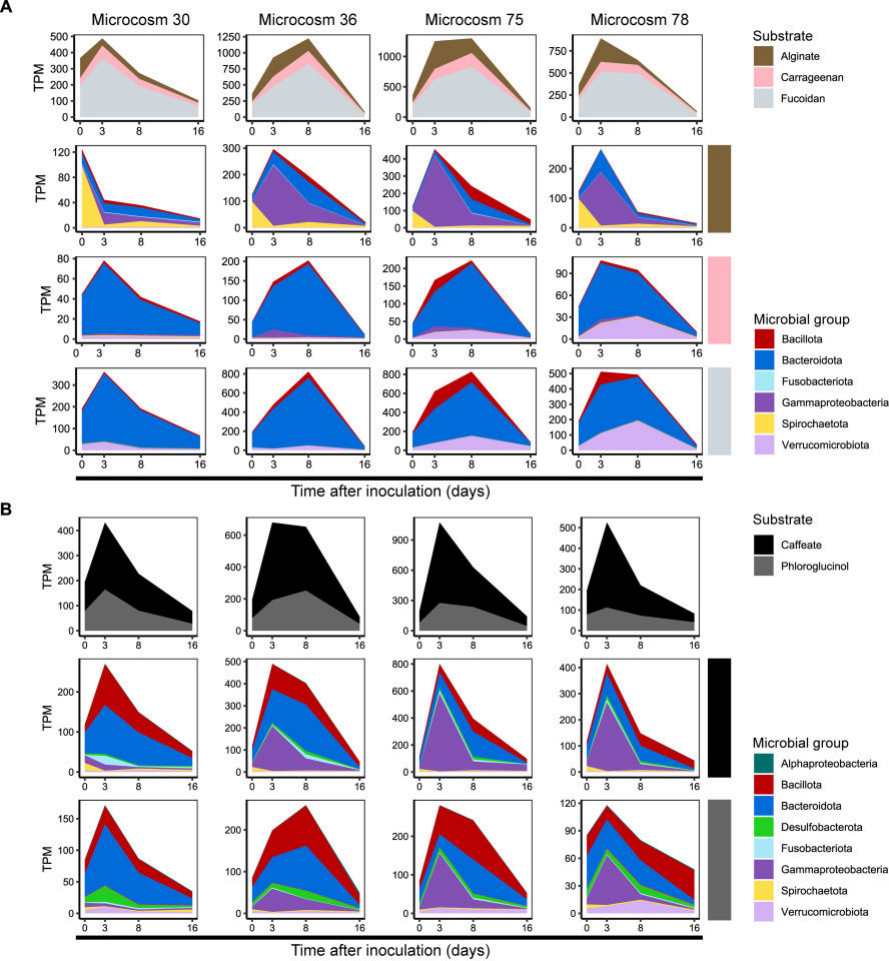
Temporal expression of microbial enzymes involved in the biotransformation of macroalgal polysaccharides and polyphenols in *K. cinerascens* microcosms. Metatranscriptomic time-series abundances are expressed as transcripts per million (TPM) for genes encoding the indicated enzyme classes. Expression of enzymes involved in the degradation of (**A**) sulfated macroalgal polysaccharides—alginate, carrageenan, and fucoidan—and (**B**) polyphenol degradation of caffeate and phloroglucinol is shown in the top rows as total transcript abundance grouped by substrate type. Subsequent rows partition the same transcript pools by the proportion contributed by each microbial phylum.

**Fig 4:**
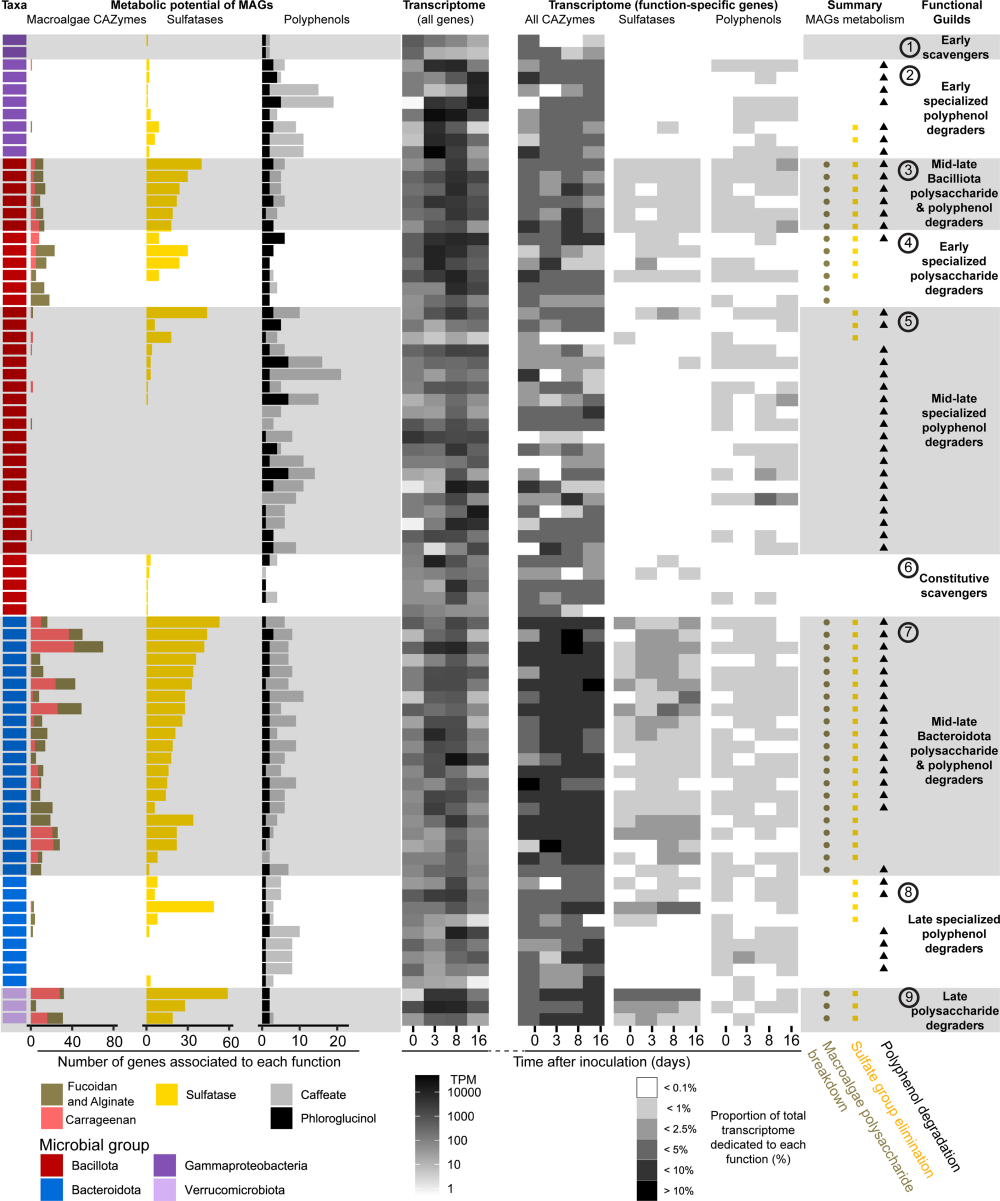
Functional guilds of the *K. cinerascens* MAGs and their transcriptomic profiles along a 16 day *in vitro* cultivation experiment. From left to right, the first column denotes the taxonomic assignment of the MAGs. The bar plots display the total gene count for CAZymes involved in the biotransformations of macroalgal polysaccharides, sulfatase genes, and genes involved in the breakdown of caffeate and phloroglucinol. Black-and-white heatmaps show the overall representation of these MAGs in the entire metatranscriptomes, then the recruitment of transcripts related to function-specific gene categories: all CAZymes, sulfatases, and genes involved in the biotransformation of caffeate and phloroglucinol. The two rightmost sets of columns provide metabolic summaries for each MAG, which were used to classify them into functional guilds within each taxon according to their capacities to biotransform and/or utilize macroalgal constituents. CAZyme categories representing the initial breakdown steps of fucoidan, alginate, and carrageenan were used to differentiate between microbes that lyse large macroalgal polysaccharides and those that scavenge on smaller oligo- and monosaccharides. The “Polysaccharide-” or “Polyphenol-degraders” guild assignment was based on the presence of macroalgae-specific CAZymes and sulfatases. In contrast, the guild assignment of “Scavenger” was based on the lack or negligible presence of these metabolic capacities. The assignation of “Early,” “Mid-Late,” or “Late” denotes when the majority of the MAGs from this guild reach the highest transcriptional proportional expression of their CAZymes (i.e., “All CAZymes” heatmap) or, in the case of Scavenger guilds, the proportional expression of all their genes was used instead (i.e., “all genes" heatmap).

The initial 1,153 MAGs obtained were de-replicated into 471 populations, and a subset of 92 dereplicated high-quality MAGs (>95% completeness, <5% contamination) from lineages expressing transcripts for enzymes targeting macroalgae constituents (Fig. 3) was further assessed regarding their metabolic potential and temporal expression of enzymes for the biotransformation of sulfated polysaccharides and polyphenols (Fig. 4). Overall, *in vitro* propagation induced an increase of transcripts related to all the polysaccharide- and polyphenol-degrading genes assessed here, which generally plummeted sometime between 8 and 16 days after inoculation (Fig. 3), potentially due to the transformation of their substrates into simpler molecules. The only exception was microcosm 30, which presented the simplest combination of macroalgae in its media among the selected microcosms. In this case, sometime between 0 and 3 days post-inoculation, the overall expression of genes related to the degradation of alginate dropped to a third of their abundance compared to levels present in the initial inoculum (Fig. 3A). Furthermore, in microcosm 30, the expression of genes related to the degradation of carrageenan and fucoidan plummeted between 3 and 8 days after its inoculation (Fig. 3A).

When comparing the microbiomes of the selected microcosms, the proportion of Gammaproteobacteria transcripts in microcosm 30 that were detected 3 days after inoculation was less than half the representation of this group in the metagenome from the same time point (Fig. 3B and C). The reduced representation of Gammaproteobacteria transcripts was also evident when comparing microcosm 30 to the other selected microcosms regarding the expression of genes involved in the degradation of alginate (Fig. 3A) and polyphenol-related enzymes (Fig. 3B).

### Identification of microbial guild structure in macroalgae processing

**Fig 5:**
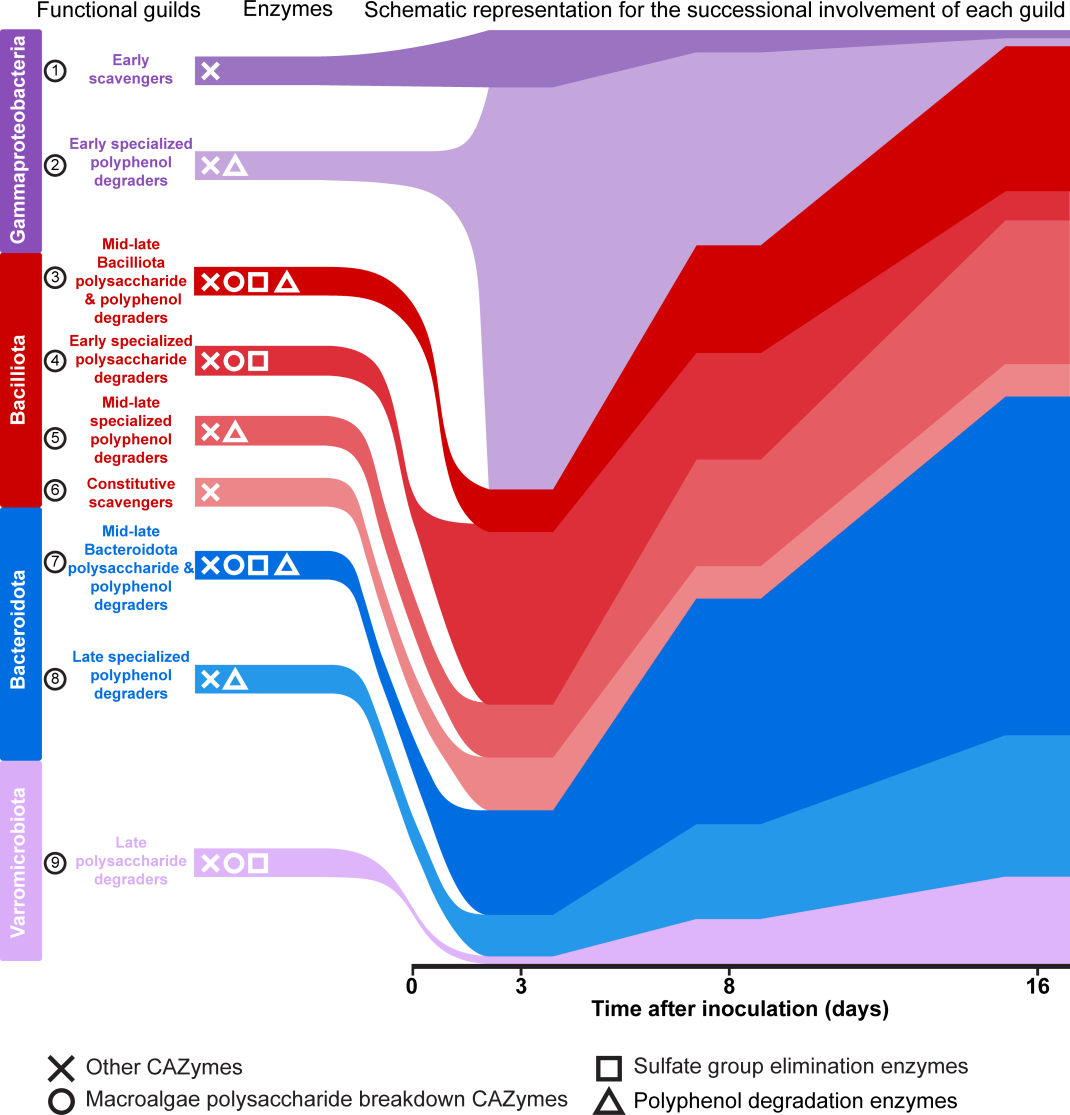
Transcriptional successional involvement of the main functional guilds involved in macroalgal biotransformations in the *K. cinerascens* 16 day *in vitro* cultivation experiments. By integrating metatranscriptomic results from the 16 day incubation experiments, this scheme illustrates the relative timing and qualitative contributions of the distinct microbial guilds described in this study to macroalgal polysaccharide and polyphenol processing and scavenging activities. Colored bands represent functional guilds grouped by taxonomic affiliation and metabolic specialization and provide a simplified, qualitative synthesis of the dominant successional patterns emerging from the transcriptomic time-series data shown in Fig. 3 and 4. Symbols adjacent to each guild indicate the primary enzyme classes expressed by that group, including CAZymes, sulfatases, and polyphenol-degradation enzymes. “Successional involvement” refers to the temporal patterns of transcriptional activity observed for these enzymes across early (days 0–3), intermediate (day 8), and late (day 16) stages of incubation.

The overall transcriptional profiles of the Gammaproteobacteria MAGs revealed the presence of two functional guilds (Fig. 4). One guild expressed enzymes involved in polyphenol degradation during the early stages of microcosm incubation, particularly before day 8, likely representing specialized populations initiating the detoxification of macroalgal polyphenols. The other Gammaproteobacteria functional guild expressed CAZyme genes associated with the degradation of the relatively bioavailable polysaccharide alginate during this same early window. The temporal confinement of these activities to the initial phase of the experiment suggests that populations from the latter Gammaproteobacteria guild operated as opportunistic scavengers and early responders utilizing readily available substrates. Thus, the two *K. cinerascens* Gammaproteobacteria functional guilds detected here significantly decrease their *in vitro* metagenomic and metatranscriptomic representation because they are likely responsible for early polyphenol degradation steps and the utilization of less complex polysaccharides (Fig. 5). This is in agreement with previous studies showing a higher proportion of Vibrionaceae among intermediate gut sections compared to the more distal hindgut communities of *K. cinerascens* (15) and other *Kyphosus* fish (17). These same studies have shown that the representation of other microbial phyla, which express the majority of the genes involved in polysaccharide breakdown and polyphenol degradation (i.e., Bacillota, Bacteroidota, and Verrucomicrobiota), increases in communities located in the more distal hindgut compared to those from intermediate gut sections of these herbivorous fish.

Transcripts assigned to the Bacillota phylum were involved in the processing of all polysaccharides and polyphenols irrespective of the medium compositions assessed here (Fig. 3). Still, the extent of their involvement was proportionally more important regarding the expression of enzymes involved in polyphenol degradation (Fig. 3B). Bacillota MAGs assigned to the genera *Faecalibacterium*, *JAKSVV01*, and *Caccovivens* showed a broader range of functional capacities and temporal expression patterns (Fig. 4). Bacillota microbes presented four functional guilds, of which the first one included members capable of both polysaccharide and polyphenol degradation, with pronounced transcriptional activity at days 8 and 16 (Fig. 4), suggesting a role in processing more structurally complex or partially transformed substrates. A second guild of Bacillota MAGs appears specialized in polysaccharide breakdown and followed an earlier transcriptional activity pattern, with higher overall transcriptional levels at days 3 and 8 (Fig. 4). The third set of Bacillota MAGs comprised a functional guild specialized in polyphenol degradation and had a higher transcriptional activity mostly at days 8 and 16 (Fig. 4). Finally, the fourth guild of Bacillota MAGs appeared to act as generalist scavengers, with no clear transcriptional pattern throughout the time series (Fig. 4). These trends suggest Bacillota include metabolically versatile taxa that are involved in distinct temporal niches within the successional dynamics of the macroalgal biotransformation process (Fig. 5).

Transcripts assigned to the Bacteroidota phylum were proportionally more important regarding the expression of enzymes for polysaccharide degradation (Fig. 3A), but were also one of the main microbial taxa involved in the processing of polyphenols irrespective of the medium compositions assessed here (Fig. 3B). MAGs from the phylum Bacteroidota were predominantly associated with the genus *Alistipes* and presented high transcriptional levels at the intermediate (day 8) and final (day 16) time points. Previous studies have reported that hindgut *Alistipes* populations from other *Kyphosus* species have the potential to utilize a wide range of macroalgal polysaccharides, thanks to the high number of CAZyme gene clusters in their genomes (7, 20). Overall, Bacteroidota MAGs with both polysaccharide and polyphenol degradation capacities exhibited elevated transcriptional activity from days 3 to 16, indicating a role in the continued degradation of polysaccharides and breakdown of polysaccharide substrates throughout the *in vitro* experiments (Fig. 5). A second Bacteroidota functional guild that seemed to specialize solely in the degradation of polyphenol had higher overall transcriptional activity towards the final time points of the experiment (Fig. 5).

Microcosms supplemented with the most complex macroalgal mixtures exhibited higher levels of Verrucomicrobiota transcripts associated with the degradation of carrageenan and fucoidan (Fig. 3A), as well as the polyphenol phloroglucinol (Fig. 3B). MAGs from the phylum Verrucomicrobiota were mostly assigned to the genus *CAKUIA01* (order Opitutales) and a high-quality MAG from the order Kiritimatiellales. These populations were primarily specialized polysaccharide degraders, with gene expression profiles indicating peak activity at the middle and final time points of the *in vitro* experiments (Fig. 5). These findings suggest that Verrucomicrobiota are particularly responsive to complex, heterogeneous mixtures of macroalgal compounds, likely benefiting from the metabolic activities of early-degrading populations and contributing to the later stages of macromolecule deconstruction.

Together, these results support the existence of a stepwise, collaborative degradation process among distinct microbial populations within the *K. cinerascens* gut microbiota. Early-acting specialists rapidly initiate the detoxification and breakdown of accessible substrates, while later-acting populations respond to the progressive transformation of more recalcitrant compounds. This ecological succession underscores the metabolic interdependence of gut symbionts in processing complex macroalgal diets.

Community composition at the metagenomic level and community functional expression patterns of the *in vitro* microcosms (Fig. 3) resembled the abundance profiles of 16S rRNA amplicon (15, 17) and community sequencing (7). These prior studies assessed the fine-scale transition observed along the natural gastrointestinal tract of herbivorous fishes, including the shift from stomach to hindgut compartments, where increasingly processed substrates support the activity of more specialized microbial taxa from the Bacteroidota, Bacillota, and Verrucomicrobiota phyla. This research further validates the ecological relevance of the *in vitro* microcosm system developed here, as well as the potential to explore additional factors relevant to the natural gut such as oxygen tolerance and microbiome stability over long timescales.

Previous metatranscriptomic studies, in the closely related *Kyphosus sydneyanus* fish (7), have shown that the biotransformations of macroalgal polysaccharides in natural samples of the two most distal sections of the hindgut are mainly performed by the Bacteroidota (class Bacteroidia), and Bacillota (classes Clostridia and Bacilli). The findings of this study build on this foundational work by expanding the identification of microbial groups that are transcriptionally active members in natural hindgut microbiomes through cDNA-based 16S rRNA gene amplicon sequencing and metatranscriptomic approaches and add a novel temporal dimension to the expression dynamics of anaerobic macroalgae-degrading microbiota using *in vitro* microcosms. These insights not only revealed when specific functional guilds are active but also how their interactions unfold over time in response to substrate availability, adding a new layer of resolution to the ecology of generalist herbivorous fish gut microbiomes. Furthermore, this is the first study assessing the biotransformation capacities of macroalgal polyphenols by complex *in vitro* microbiomes that closely resemble the natural hindgut microbiome of generalist herbivorous fish. However, because phloroglucinol is only one of the final breakdown products of diverse polyphenols, the degradation of macroalgal polyphenols likely involves many additional enzymes beyond those assessed here. As a result, our evaluation of polyphenol-related enzyme expression was constrained by the current range of annotated targets available in the CAMPER database (10). Thus, future studies identifying enzymes with validated capacities to biotransform physiologically relevant macroalgal polyphenol substrates (2) and identifying their gene sequence will be vital to developing much-needed bioinformatic strategies to identify these enzymes.

Despite being informed by key physicochemical features of the teleost hindgut, this *in vitro* microcosm approach necessarily represents a simplified approximation of the natural gut environment. Our experimental design incorporated the primary short-chain fatty acids (SCFA) typically reported for fish intestines (i.e., acetate, propionate, and butyrate) (21, 22) as well as type II mucins (23) and bile salts (24) known to structure teleost gut microbiota. However, the native hindgut environment is considerably more complex, characterized by dynamic SCFA gradients, structurally and chemically diverse host- and diet-derived polysaccharides, continuous epithelial turnover, immune surveillance, and interactions with parasites, viruses, and other microbial predators that were not replicated in these closed systems (25). Consequently, selective pressures shaping microbial activity, spatial organization, and cross-feeding interactions *in vivo* are only partially captured by these microcosms. Nevertheless, the cultures retained substantial taxonomic fidelity to the source communities, propagating up to 96%–99% of all ASVs assignable at the family level (Fig. S4). Together, these results suggest that although *in vitro* microcosms cannot fully emulate the ecological and physiological complexity of the fish hindgut, they effectively preserve the dominant functional guilds likely responsible for macroalgal biotransformation processes.

## MATERIALS AND METHODS

### Fish sampling

*Kyphosus cinerascens* specimens were collected by local fishers using a spear gun directly offshore of the Ocean Era facility at Keahole Point, Kona, HI, USA, on 7 July 2022 (19.7286, −156.0619). Biometrics such as mass and fork length (the distance between the snout and the fork of the tail fin) were measured for each fish (Table S1). Specimens were dissected as described previously (15) to collect the microbiota from the distal end (hindgut) section of the host intestine under anaerobic conditions.

### Propagation of fish gut microbiota

To generate medium combinations (Fig. S1; Table S2) that mimicked the dietary elements of the *Kyphosid* fish host (13–15), macroalgae *Turbinaria* spp., and *Dictyota* spp. were collected from Keahole Point (Kona, HI, USA; 19.7286, −156.0619), while *Ulva* spp., *Halidrys* spp., and assorted coralline macroalgae were collected from La Jolla Tide Pools (San Diego, CA, USA; 32.841566, −117.281807). *Agardhiella subulata* red macroalgae were cultivated on land (26) at the Ocean Era facilities (Kona Island, HI, USA). Seawater previously passed through a 0.22 µm pore size filter (Sterivex, Millipore) was used to soak and triple-wash the macroalgae—to remove sand, epibionts, and macrofauna—before being blended and freeze-dried into a fine powder. The artificial seawater (ASW) of 35 practical salinity units used here consisted of Instant Ocean, 40 g/L (Spectrum Brands, Blacksburg, VA). Additionally, a basal salts medium (BSM) was formulated to replicate the natural physico-chemical parameters of marine bony fish gastrointestinal tracts (see “Anaerobic *in vitro* microcosms to propagate *K. cinerascens* gut microbiota”). Resazurin was added at 0.005 g/L final concentration to visually examine O_2_ levels of all cultures. A total of 4 g/L of freeze-dried macroalgae combinations were added to each culture (Fig. S1 and Table S2). Since the microbiota residing in the distal intestine section (hindgut) of *Kyphosus* fish have the highest proportion of genes involved in the biotransformation of macroalgae (5), the hindgut contents of six *K. cinerascens* fish were combined to serve as inoculum for all the microcosms of this study. The gut inoculum was mixed with anaerobic BSM and sieved inside the anaerobic chamber through a sterile 1 mm strainer to remove macroalga pieces that could alter the medium composition. Microcosms were inoculated with the sieved *K. cinerascens* hindgut content mixtures at a final 1:180 dilution ratio in 50 mL–100 mL serum bottles crimp-sealed with a rubber septum. The 50 mL of headspace was flushed with up to ~29 PSI with N_2_:CO_2_ 80:20 gas. All cultures were maintained at 22°C during the entire experiment. When sampling each time point, the exposed surface of the serum bottle crimper and rubber stopper were sterilized with alcohol pads before retrieving 1 mL of each culture. These samples were mixed with 1 mL of 2× DNA/RNA Shield solution (Zymo) and then stored at −80°C until further processing. One hundred fifty microliters of each natural sample, culture, and the non-inoculated medium controls was aliquoted into 96-well plates to screen their microbiomes.

### Anaerobic *in vitro* microcosms to propagate *K. cinerascens* gut microbiota

A BSM was formulated to replicate the natural physico-chemical parameters of marine bony fish gastrointestinal tracts regarding the overall drop in Na^+^ plus Cl^-^ to the bloodstream through the GI-tract epithelium. This decreases the concentrations of these ions from >300 mM in seawater to 50 mM–80 mM in the gastrointestinal-tract lumen while establishing high concentrations of Mg^2+^, SO_4_^2-^, and the saturation of carbonate (27). Some microcosms were also amended with marine bony fish type II mucins (23), bile salts (24), and SCFAs that have been previously detected in *Kyphosus* hindguts (21, 22) to favor the propagation of microbial groups that might require these nutrients (6).

Specifically, BSM was a mixed solution of: MgSO_4_, 10.833 g/L; K_2_CO_3_, 0.8293 g/L; CaCO_3_, 0.6 g/L; MgCO_3_, 1.6863 g/L; and a 1:10 dilution of ASW. Cysteine hydrochloride monohydrate (1028390100, Sigma) 0.5 g/L final concentration was added before autoclaving to the BSM to make it anaerobic. The following components were added to make different culture medium combinations (Fig. S1 and Table S2): 3.62 g/L mucin type II (M2378-100G, Sigma) (23); a final concentration of 0.226 g/L taurocholic acid sodium salt hydrate (T4009-5G, Sigma) and chenodeoxycholic acid (C9377-5G, Sigma) at a 4:1 ratio (23); biotin, 2 mg/L (Sigma); folic acid, 2 mg/L (Sigma); pyridoxine, 10 mg/L (Sigma); riboflavin, 5 mg/L (Sigma); thiamine hydrochloride, 5 mg/L (Sigma); cyanocobalamin, 0.1 mg/L (Sigma); nicotinic acid, 5 mg/L (Sigma); p-aminobenzoic acid, 5 mg/L (Sigma); lipoic acid, 5 mg/L (Sigma); DL-pantothenic acid, 5 mg/L (Sigma).

### DNA “Fast-Extract” and DNA-based 16S rRNA gene amplicon sequencing

A high-throughput and inexpensive “Fast-Extract” DNA extraction procedure was implemented here to process the hundreds of microcosms and the natural *K. cinerascens* hindgut samples. The titration of a mixed culture of *Bacillus subtilis* and *Paracoccus* spp. with known cell concentrations revealed that the absolute limit of detection for the Fast-Extract procedure coupled with 16S rRNA amplicon sequencing was between 31 and 16 cells (Fig. S1). Still, the number of reads assigned to *B. subtilis* and *Paracoccus* spp. fell within the same range as the background noise (i.e., quality filtered reads assigned to taxa that were not present in the initial mixed culture) when <1,600 cells were present in the biological sample (Fig. S1). Since >90% of the reads associated with the background noise were assigned to the Vibrionaceae family (i.e., from either the *Catenococcus* or *Vibrio* genus; Table S3) the observed sequence background noise is likely the result of well-to-well contamination (28) with neighboring microcosm samples processed for nucleic acid extraction in the same 96-well multiplates and sequenced in parallel (Fig. 1B).

For the Fast-Extract procedure, cell lysis of microcosm aliquots of 150 µL was performed using a heat and freeze method. Specifically, aliquots were heated to 95°C for 10 min and then immediately placed at −20°C for another 10 min. The samples were then placed on ice and thawed before being centrifuged at 2,000 × *g* for 5 min at 4°C. Immediately after centrifugation, 100 µL of supernatant was transferred to a new 96-well plate to undergo a 1× bead cleanup step using AMPure XP beads (Beckman Coulter). Briefly, the magnetic beads were allowed to equilibrate to room temperature and then vortexed 30 s at high speed before adding 1 volume (100 µL) to each sample. The beads and samples were mixed by vortexing for 5 s at the highest speed and left to incubate at room temperature for 5 min. The plate was placed on a magnetic rack, and the supernatant was discarded to remove cellular debris. While on the magnetic stand or plate, samples were washed twice with two volumes of freshly made 70% ethanol (vol/vol), allowing for the beads to incubate for 30 s with this solution before removing it. Then the magnetic beads were air dried for 5 min at room temperature before removing them from the magnetic rack and gently resuspending them in 30 µL of sterile DNase-free water. The samples were left to incubate for 10 min at 37°C before the beads were decanted on a magnetic rack and the supernatant with the DNA was retrieved and stored at −20°C for further processing. A variety of positive and negative controls were included in the DNA extraction steps. Positive controls were utilized to estimate the limit of detection of the assay and assess or ensure minimal contamination occurred following the Katharoseq protocol (28–30). Seven serial dilutions of a *Paracoccus* spp. and *Bacillus subtilis* sp. mixed culture with an initial concentration of 1.6 × 10^9^ and 3.1 × 10^8^ cells/mL, respectively, were subjected to the same Fast-Extract procedure and 16S rRNA amplicon sequencing described above to compare input cell counts with the resulting sample composition to determine the absolute limit of detection of this procedure. Cell concentrations for the initial *Paracoccus* spp. and *Bacillus subtilis* sp. mixed culture were corroborated by flow cytometry as previously described (28). The V4 region (∼290 bp) of the 16S rRNA gene was amplified using a two-step PCR protocol to create dual-barcoded amplicons. The first reaction used primers 515F-Y and 806rb with overhangs for attachment of Illumina-compatible indexes in the second reaction (31). The initial reaction was performed in triplicate using a high-fidelity Q5 polymerase (NEB, Ipswich, MA) with the following steps: initial denaturation of 30 s at 98°C; 25 cycles of 10 s at 98°C, 20 s at 50°C, 30 s at 72°C; final extension of 2 min at 72°C. The second reaction was performed as described above except the amplification steps consisted of only eight cycles with an annealing temperature of 56°C. Barcoded amplicons were cleaned using AMPure XP beads (Beckman Coulter, Brea, CA), pooled at equimolar concentrations, and sequenced using Sp500 chemistry (2 × 250) on a MiSeq instrument (Illumina, San Diego, USA).

### Parallel DNA and RNA extractions and generation of cDNA

A 2 mL sample of the natural hindgut contents of each *Kyphosus* fish, as well as composite mixtures of all six fish hindgut samples (used to inoculate cultures), were preserved by mixing in three volumes of DNA/RNA Shield solution (Zymo), then stored at −80°C until further processing. These natural samples and a select number of cultures were processed for simultaneous parallel extraction of nucleic acids from the same biomaterial using the DNA and RNA Purification protocol of the ZymoBIOMICS MagBead DNA/RNA kit (Zymo). Samples were processed according to the manufacturer’s instructions, except that 750 µL of biomaterial was used in each extraction, with the volume of new DNA/RNA Shield solution added initially to each bead-beating tube reduced to 350 µL. Other modifications to the manufacturer’s recommended procedure were that all samples were bead-beaten for 40 min at 4°C using a Vortex-Genie 2, and the incubation with Proteinase K was skipped. Immediately after extraction, 11 µL of RNA solution from the natural hindgut contents of each *Kyphosus* fish and four replicate samples of the inoculum mixture of all six fish hindgut contents was retro-transcribed into cDNA with the SuperScript III kit (Invitrogen) following the random primer procedure.

### 16S rRNA gene amplicon sequence analysis

All amplicon sequence data were analyzed together using the DADA2 package (v.1.11.3) (32) implemented in R (v.3.4.4) (33). The primers were removed using CutAdapt (v.1.2.1) (34), and the sequences from each pair were trimmed and quality-filtered (maxN = 0, maxEE = c(2,2), truncQ = 2, rm.phix = TRUE, trimLeft = 10, trimRight = 20, minLen = 160). ASVs were inferred from de-replicated sequences. Taxonomic assignment was performed using the Genome Taxonomy Database (GTDB v.202) for Bacteria and Archaea (35). Chimeras were removed with “removeBimeraDenovo” using the “consensus” removal method. All sequences assigned to mitochondria or chloroplasts were removed, and DECONTAM (36) was used to further identify contaminants within these data sets using the “frequency” method with a default probability threshold (TRUE if *P* < 0.1) to discriminate foreign taxa observed in the negative controls of autoclaved medium constituents without hindgut inoculum (i.e., microcosm numbers 1 to 15). Samples from these negative controls and those with fewer than 1,000 reads were excluded, leaving a total of 23 natural hindgut samples (11 from the DNA and 12 from the cDNA fractions) and 172 microcosm samples. Differences in taxonomic abundance between DNA and cDNA samples were calculated using Tukey’s honestly significant difference test at *P* < 0.05 (37). The proportion of ASVs in each sample was plotted with the Phyloseq package (38). Alpha diversity was evaluated using Breakaway (39) with unaltered frequency counts of quality-filtered and decontaminated ASVs.

We used the ASV count table to construct a network of samples (ANET-samples), applying the Association Network (Anets) framework to assess associations between pairs of samples based on the similarity of their ASV co-occurrence patterns (40). Briefly, the ASV table was transformed into a matrix where both rows and columns represent samples, and each cell contains the number of shared ASVs between each sample pair. To quantify similarity, we calculated the Pearson correlation coefficient for each pair of samples. Pairs with a correlation coefficient (*R*) ≥ 0.70 were considered associated. The resulting correlation matrix was converted into a Cytoscape-compatible data set using R, and sample networks were visualized in Cytoscape v.3.6.1 (https://cytoscape.org) (41, 42). Modularity of the sample network was assessed using MCL assignments based on correlation thresholds of *R* ≥ 0.70. In addition, ASV interaction networks were inferred using FlashWeave with the “sensitive” option enabled (43). FlashWeave applies a centered log-ratio transformation to account for compositional data characteristics. Modularity in ASV networks was also computed via MCL clustering, using the same correlation threshold (*R* ≥ 0.70) to define modules. All statistical analyses and visualizations of the amplicon sequencing data were conducted using the Microeco package (v.0.20.0) (44) in R version 4.5.0.

### Metagenomic and metatranscriptomic sequencing

Barcode-indexed sequencing libraries were generated from the metagenomic DNA samples using the DNA KAPA Hyper Prep Kit (Kapa Biosystems-Roche, Basel, Switzerland) following the Earth Microbiome Project protocol (45). The pools were quantified by qPCR with a Kapa Library Quant kit (Kapa Biosystems-Roche, Basel, Switzerland), and each pool was sequenced using 2 × 150 chemistry on a NovaSeq instrument (Illumina, San Diego, CA, USA). Total RNA (250 ng) was subjected to the depletion of ribosomal sequences using the Qiagen QIAseq FastSelect kit following the manufacturer’s instructions (Qiagen, QIAseq FastSelect –rRNA Fish Kit combined with the 5S/16S/23S rRNA Kit). The ribo-depleted RNAs were then used to generate strand-specific and barcode-indexed RNA-seq libraries using the KAPA RNA HyperPrep Kit (Kapa Biosystems-Roche, Basel, Switzerland) following the instructions of the manufacturer. The fragment size distribution of the RNA libraries was verified via micro-capillary gel electrophoresis on a Bioanalyzer 2100 (Agilent, Santa Clara, CA, USA), quantified by fluorometry on a Qubit fluorometer (Life Technologies, Carlsbad, CA, USA), and pooled in equimolar ratios that were sequenced using 2 × 150 chemistry on a NovaSeq instrument (Illumina, San Diego, CA, USA).

### Metagenomic and metatranscriptomic assembly and bioinformatic processing

Metagenomic reads were cleaned with fastp v.0.23.4 (46) with --trim_poly_g, and assembled with metaSPAdes v.3.15.5 (47), and annotated with Prokka v.1.14.6 (48). Metagenomic binning was performed with COMEBin v.1.0.3 (49). MAG quality was assessed with CheckM2 v.1.0.2 (50), and taxonomic lineage was assessed with GTDB-Tk v.2.3.2 (51) using Genome Taxonomy Database release 214 (52). MAGs were dereplicated with dRep v.3.4.2 using default parameters (53). A phylogenetic tree of MAGs was generated using PhyloPhlan v.3.1.68 (54) and visualized with the ggtree v.3.4.0 package (55) in the R v.3.6.1 environment. The taxonomic distribution of metagenomic and metatranscriptomic reads was determined using Kraken v.2.1.3 (56) with a custom database utilizing all sequences in NCBI nr (57) as of August 2022. Kraken outputs were converted to biom data files using kraken-biom (https://github.com/smdabdoub/kraken-biom) and then frequency and taxonomy tables were generated with “qiime tools extract” in the Qiime2 environment (58). The metagenomic read-based community analyses were then performed using the same procedures for amplicon frequency and taxonomy data tables as described above.

Metatranscriptomic reads were cleaned with fastp v.0.23.4 (46) with the settings --trim_poly_g -f 10 -t 2. All rRNA sequences that remained were computationally removed using KneadData v.0.10 (59). Individual *de novo* metatranscriptomes were assembled using rnaSPAdes v.3.15.5 (60), and transcripts of interest were selected using TransDecoder v.5.7.1 (https://github.com/TransDecoder/TransDecoder).

### Gene annotation and expression

Sequences associated with CAZyme genes were annotated using dbCAN v.4.1.4 (61) based on results from DIAMOND (62) and HMMER (63). Similarly, sulfatase gene sequences were annotated using hidden Markov models provided by the SulfAtlas v.2.3.1 database (64), and genes for enzymes targeting polyphenols were annotated using CAMPER v.1.0.0 (10).

The taxonomic assignment of the metagenomic and metatranscriptomic reads was done as described above, and gene expression values in TPM were calculated using RSEM v.1.3.3 (65) by mapping them to MAGs assembled in this study using Bowtie v.2.5.4 (66).

## Data Availability

Sequence reads are available under SRA BioProject PRJNA1404287 and BioSamples SAMN54710122–SAMN54710453. Assembled metagenomes, binned MAGs, and predicted proteins for binned MAGs are available on Zenodo (https://zenodo.org/) under DOI no. 10.5281/zenodo.18273293.

## SUPPLEMENTAL MATERIAL

Supplemental Figure Legends mbio.03382-25-s0001.docx Legends for Fig. S1-S6.

Figure S1 mbio.03382-25-s0002.tif Experimental design of *K. cinerascens* hindgut microcosms and their validation.

Figure S2 mbio.03382-25-s0003.tif Fast-extract limit of detection for 16S rRNA amplicon sequencing.

Figure S3 mbio.03382-25-s0004.tif Breakaway diversity estimate for the *K. cinerascens* natural hindgut microbiome and its propagated *in vitro* communities.

Figure S4 mbio.03382-25-s0005.tif Composition of the *K. cinerascens* natural hindgut microbiome, at the family level, showing microcosms that propagated the largest number of microbial families.

Figure S5 mbio.03382-25-s0006.tif ASVs differentially represented in the *K. cinerascens* natural hindgut microbiome and the communities propagated through its microcosms.

Figure S6 mbio.03382-25-s0007.tif Phylogeny and biological origin for the initial (non-dereplicated) MAGs generated in this study for the main microbial groups comprising the *K. cinerascens* hindgut microbiome.

Supplemental Tables mbio.03382-25-s0008.xlsx Tables S1-S7.

ASM does not own the copyrights to Supplemental Material that may be linked to, or accessed through, an article. The authors have granted ASM a non-exclusive, world-wide license to publish the Supplemental Material files. Please contact the corresponding author directly for reuse.
