# Supplementary material for: Detoxifying and depolymerizing microorganisms reveal intertwined guild collaborations in the gut microbiome of the generalist macro-algivorous fish Kyphosus cinerascens
Source: mBio. 2026 Jun 24;17(8):e03382-25. doi: 10.1128/mbio.03382-25 (PMC13463842; doi:10.1128/mbio.03382-25)
Supplement: Supplementary file 1 [file mbio.03382-25-s0001.docx]

**SUPPLEMENTARY FIGURES**

**FIG S1** Experimental design of *K.* *cinerascens* hindgut microcosms and their validation. The proportion of each macroalga used in the culture media is shown in parentheses. Vitamin concentrations are detailed in the Materials and Methods section. Marine fish bile salts were used at a final concentration of 0.226 g/L. The hindgut inoculum final dilution ratio corresponds to the wet weight dilution factor of the natural sample mix in each microcosm. A “microcosm identifier number” was given to both negative controls that contained only sterilized components (microcosms number 1-15) and experimental cultures that were inoculated with the same hindgut inoculum (microcosms number 16-80).

**FIG S2** Fast-Extract limit of detection for 16S rRNA amplicon sequencing. Quality-filtered reads assigned to *Paracoccus* spp., *B. subtilis* sp. throughout a dilution series with known cell numbers are compared to those assigned to taxa that were not present in the initial biological material processed through this DNA extraction method.

**FIG S3** Breakaway diversity estimate for the *Kyphosus cinerascens* natural hindgut microbiome and its propagated *in vitro* communities. Source DNA (n=11) and cDNA (n=12) samples are shown after incubation at day 3 (T03, n=62), 8 (T08, n=59), and 16 (T16, n=52) are shown. Diversity estimates are based on all the ASVs present in each of the samples after quality filtering and removal of contaminants (mitochondria, chloroplasts, and contaminant ASVs detected in negative controls of autoclaved media constituents without hindgut inoculum) and artificial sequences (*i.e*., chimeras).

**FIG S4** Composition of the *Kyphosus cinerascens* natural hindgut microbiome, at the family level, showing microcosms that propagated the largest number of microbial families. The upper and lower schematics depict the presence/absence and proportional abundance, respectively, of microbial families comprising >5x10^-4^ % of the total ASVs in at least one sample of the natural *K. cinerascens* hindgut active microbiome (consisting of up to two replicates ⎼“rep_1” and “rep_2”⎼ per sampled specimen which were numbered from 1 to 6; Table S1) either within the DNA (n=11) or cDNA fractions (n=12). Microbial community composition is shown for microcosm media composition numbers 30, 35, 75, and 78 (Fig. S1 and Table S2) sampled at days 3 (T03), 8 (T08), and 16 (T16). The graph at the bottom of the figure represents the proportion of each taxon, considering all ASVs that were taxonomically assignable at the family level. “NA” denotes a time point that was discarded because it had <1,000 total sequences assigned at the family level.

**FIG S5** ASVs differentially represented in the *Kyphosus cinerascens* natural hindgut microbiome and the communities propagated through its microcosms. (A) The relative abundance of each of the top 28 ASV modules (Table S7) within each sample as determined through a correlation analysis network, grouped using Markov Cluster Algorithm (MCL). Modules comprise ASVs present in at least 5 samples and present a significant (corr > 0.7) co‐association. The network was transformed to consider that edges are all positive. (B) Redundancy Analysis (RDA) to show how the media components (black arrows) explain the observed variation in the samples (dots). Colors denote the samples with regards to each MCL cluster. Ellipsoids are defined by a 95% confidence interval. The clustering of microcosm #78 at eight and sixteen days after inoculation are denoted in both sections of the figure to showcase their similarity to cDNA and DNA microbiota profiles from natural fish samples grouped within clusters one, two, and three.

**FIG S6** Phylogeny and biological origin for the initial (non-dereplicated) MAGs generated in this study for the main microbial groups comprising the *K.* *cinerascens* hindgut microbiome.
